# Identifying future zoonotic disease threats: Where are the gaps in our understanding of primate infectious diseases?

**DOI:** 10.1093/emph/eot001

**Published:** 2013-01-22

**Authors:** Natalie Cooper, Charles L. Nunn

**Affiliations:** ^1^School of Natural Sciences; ^2^Trinity Centre for Biodiversity Research, Trinity College Dublin, Dublin 2, Ireland and ^3^Department of Human Evolutionary Biology, Harvard University, Cambridge, MA 02138, USA.

**Keywords:** sampling events, parasite species richness, Global Mammal Parasite Database, relative sampling effort

## Abstract

**Background and objectives:** Emerging infectious diseases often originate in wildlife, making it important to identify infectious agents in wild populations. It is widely acknowledged that wild animals are incompletely sampled for infectious agents, especially in developing countries, but it is unclear how much more sampling is needed, and where that effort should focus in terms of host species and geographic locations. Here, we identify these gaps in primate parasites, many of which have already emerged as threats to human health.

**Methodology:** We obtained primate host–parasite records and other variables from existing databases. We then investigated sampling effort within primates relative to their geographic range size, and within countries relative to their primate species richness. We used generalized linear models, controlling for phylogenetic or spatial autocorrelation, to model variation in sampling effort across primates and countries. Finally, we used species richness estimators to extrapolate parasite species richness.

**Results:** We found uneven sampling effort within all primate groups and continents. Sampling effort among primates was influenced by their geographic range size and substrate use, with terrestrial species receiving more sampling. Our parasite species richness estimates suggested that, among the best sampled primates and countries, almost half of primate parasites remain to be sampled; for most primate hosts, the situation is much worse.

**Conclusions and implications:** Sampling effort for primate parasites is uneven and low. The sobering message is that we know little about even the best studied primates, and even less regarding the spatial and temporal distribution of parasitism within species.

## Abstract (precis)

Across all primates, less than two thirds of primate viruses, protozoa and helminths have currently been recorded. Similar effects emerge in analyses of parasite sampling by country, and for individual primate species. Therefore a great deal more disease sampling is needed to understand zoonotic disease risks to humans.

## INTRODUCTION

Many of the most devastating infectious diseases in humans have origins in wildlife [1–3]. For example, the global AIDS pandemic originated through human contact with wild African primates [4] and influenza viruses circulate among wild bird populations [5]. These are not only historical occurrences. Recently, for example, rodents were identified as the source of a hantavirus outbreak in Yosemite National Park, USA [6] and a novel rhabdovirus (Bas-Congo virus) of probable animal origin emerged in the Democratic Republic of Congo [7]. As human populations continue to expand into new areas and global changes in temperature and habitat alter the distributions of wild animals, humans around the world will have greater contact with wildlife [8]. Thus, understanding which infectious agents have the potential to spread from animals to humans is crucial for preventing future human disease outbreaks. Here, we outline current gaps in our knowledge of primate infectious diseases at phylogenetic and geographic scales. By doing so, we provide new directions for sampling wild primates and a statistical framework to address this issue in other groups.

The first step in predicting zoonotic disease risks to humans is to identify the animal hosts of infectious agents. This information provides several insights. First, it gives information on the host range and specificity of the infectious agent. Second, it provides information on the geographic distribution of the infectious agent in wildlife, which can be compared with human population density. Finally, knowing the hosts of an infectious agent also provides information on risks for host shifts to humans [9, 10]. For example, a host living at high density is likely to exhibit higher prevalence of the infectious disease and to have more contact with humans or domesticated animals.

Many efforts are being made to document and collate information on wildlife and human diseases (e.g. HealthMap [11], EID Event Database [2] and Global Mammal Parasite Database (GMPD) [12]). Unfortunately, large-scale analyses of this type have revealed major variations in sampling effort among hosts and geographic regions, with some species and areas being sampled rarely or not at all [10, 13]. If we hope to use wildlife disease data to make reliable predictions about future risks to humans, we need to increase sampling in potential hosts and the areas in which they are found. However, before we can do this, we need to identify gaps in our knowledge of wildlife infectious diseases.

Here, we investigate gaps in our knowledge of primate parasites. We chose primates because they are our closest relatives, and partly as a consequence, many of humanity’s biggest killers have originated in wild primates (e.g. HIV [4]). In addition, much is known about primate parasites. We acknowledge at the outset, however, that many other vertebrates have been sources of emerging infectious diseases in humans, and are thus suitable for extensions of the effort conducted here. We use the word parasite in a general sense, referring to both microparasites such as viruses, bacteria, fungi and protozoa, and macroparasites such as helminths and arthropods. To assess gaps in our understanding of primate parasites, we examined records from the GMPD [12]—a large-scale compilation of parasite records from wild mammals—and use these data to quantify and model variation in sampling effort.

## METHODOLOGY

### Data collection

We obtained host–parasite records from the GMPD (accessed 15 October 2012; [12]), geographic range maps from IUCN [14] and the dated consensus phylogeny from ‘10kTrees’ version 3 [15]. For consistency across our analyses, we only included primate species found in both the range maps and phylogeny, and that we could identify to the species level using the taxonomy of Wilson and Reeder [16]. For analyses of geographical sampling gaps, we obtained latitude and longitude coordinates for each host–parasite record with locality data from the GMPD.

For each primate species, we collated data on adult body mass (g) from Jones *et al.* [17]. We also defined the substrate use of each species as terrestrial (>90% of time on ground), semi-terrestrial (<90% but >50% of time on ground), semi-arboreal (<90% but >50% of time in trees) or arboreal (>90% of time in trees) using Nowak [18], and treated this as a continuously varying character in the analyses. For each country, we assembled data on gross domestic product (GDP) per capita (USD), land area (km^2^), and the number of airports from Central Intelligence Agency [19] and Emerson *et al.* [20]. We estimated airport density (airport/km^2^) by dividing the number of airports by the land area of the country.

Overall, our dataset contained 228 primate species from 89 countries. We located host–parasite data for 166 of these species from 57 countries. The remaining 62 species and 32 countries have no records in the GMPD and were listed as ‘unsampled’ in our analyses. Our parasite data contain 651 unique parasite species or genera with non-zero prevalence in primates (87 arthropods, 50 bacteria, 6 fungi, 326 helminths, 115 protozoa and 67 viruses). Our dataset also contains 46 unique parasite species or genera that have been looked for in primates but never found (these data are important for estimating sampling effort, see below). We included all parasites when quantifying the relative amount of sampling by species and country, even those we could only identify to the generic level; for species accumulation curves, however, we only included parasites we could identify to species or strain to avoid double counting parasite species. This left us with 161 primate species and 502 parasite species with non-zero prevalence in primates (73 arthropods, 32 bacteria, 4 fungi, 242 helminths, 93 protozoa and 58 viruses) for these analyses. We also excluded 22 ‘unsampled’ primates from our models of variation in sampling effort among primate species because we were unable to locate life history data for them.

#### Sampling effort

Our measure of sampling effort is the number of sampling events for each primate. We define a sampling event as one primate species being sampled for one parasite species in one location in one paper. The number of sampling events in a paper depends on how the results were reported in the paper, and hence how they were added to the GMPD. For example, a paper reporting that *Pan troglodytes* is infected by *Ascaris lumbricoides* represents one sampling event; a paper reporting that *P. troglodytes* is infected by *A. lumbricoides* and SIVcpz in Location A and Location B represents four sampling events. This method assumes that each sampling event represents equivalent research effort; however, some sampling events may represent multiple years, multiple populations and/or multiple individuals, while others represent only one individual sampled once. Other samples may be counted multiple times, for example one fecal sample may reveal several parasites. However, in general, we believe that our definition of sampling events should give us a conservative estimate of sampling effort. Note that we included sampling events with zero prevalence for the parasite sampled because these still represent valid sampling effort.

In total, our host–parasite data consisted of 5459 sampling events, which we used to quantify relative sampling of primate species. Of these sampling events, 4067 have georeferenced localities in the GMPD and thus we also used these to quantify relative sampling of geographic regions. As mentioned above, we only included parasites we could identify to species or strain for species accumulation curves, leaving us with 3999 sampling events in these analyses. These criteria meant our species accumulation curves only use around 75% of our sampling events for some analyses, but they are necessary to ensure that we are using the highest quality data in the analyses of specific areas. It also further highlights the need for more research into primate parasites. We deposited all data in the Dryad repository: doi:10.5061/dryad.510sb.

### Analyses

#### Variation in sampling effort among primate species

All else being equal, primates should be sampled in proportion to their abundance, so we used ln(geographic range size) as a proxy for abundance and assumed primates with the largest geographic range sizes should be sampled more than primates with small ranges. We estimated sampling relative to geographic range size using the residuals from a phylogenetic generalized least squares (PGLS) model of ln(sampling events) against ln(geographic range size), fitted using the R package caper [21] (Appendix 1). We considered primates with positive residuals as being relatively better sampled given their geographic range size than primates with negative residuals, and displayed these results on the phylogeny. We also expect great apes (Hominoidea) to be better sampled than other primates, so we tested this using phylogenetic analysis of variance (ANOVA; Appendix 1).

#### Variation in sampling effort among geographic regions

We assumed that countries should be sampled in proportion to the number of primates found within the country, i.e. countries with high primate species richness should be sampled more than countries with low primate species richness. We therefore estimated sampling relative to primate species richness within each country using the residuals from a spatial generalized least squares (GLS) model of ln(sampling events) against ln(primate species richness) using the R package nlme [22] (Supplementary Data, Appendix 1). We considered countries with positive residuals as relatively better sampled given their primate species richness than countries with negative residuals and displayed these results on a world map.

#### Modeling variation in sampling effort among primate species

We predicted that the following variables would influence sampling effort among primate species: (i) geographic range size (we expect primates with larger geographic ranges to be sampled more often than primates with smaller ranges); (ii) phylogenetic distance from humans (medical research is likely to focus on our closest relatives, thus we expect them to be sampled more often); (iii) body size (small species are easier to capture and so likely to be sampled more than larger species) and (iv) substrate use (terrestrial species are easier to sample than arboreal species and thus should be sampled more often). We therefore fit the following model:
(1)
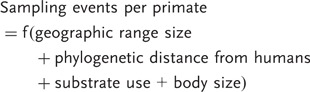

We fit PGLS models for the 205 primate species for which we had data (including 40 ‘unsampled’ primates). All variables except substrate use were natural log transformed prior to analysis. We also used caper [21] to estimate phylogenetic signal (i.e. *λ*, Supplementary Data, Appendix 1) in the number of sampling events across primates. Phylogenetic signal is the tendency for related species to resemble each other more than they resemble species drawn at random from a phylogenetic tree [23]. High phylogenetic signal, i.e. *λ* values close to 1, indicates that closely related species have similar numbers of sampling events, whereas low phylogenetic signal, i.e. *λ* values close to 0, indicates that the number of sampling events varies randomly across the phylogeny. We acknowledge that many of our variables—such as sampling effort and geographic range size—are not biological traits subject to normal evolutionary change. However, they may still show phylogenetic non-independence, and *λ* enables quantification of that non-independence regardless of which underlying process generates it.

#### Modeling geographic variation in sampling effort

We predicted that the following variables would influence sampling effort among countries: (i) primate species richness (countries with more primates are likely to be sampled more often than countries with fewer primates because there are more primates to sample); (ii) GDP (we expect countries with a high GDP to have more resources for disease monitoring and hence to be sampled more often than countries with a lower GDP) and (iii) airport density (countries with more airports given their area are likely to be easier to visit, and hence disease monitoring should be more frequent). We therefore fit the following model:
(2)
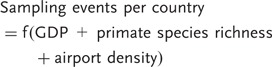

We fit spatial GLS models for the 89 countries that contain primates (including 32 ‘unsampled’ countries). All variables were natural log transformed prior to analysis. Note that the results were almost identical when using a spherical rather than an exponential correlation structure, so we only report the exponential correlation structure results.

#### Extrapolating parasite species richness for primates and countries

We first used the R package vegan [24] to plot species accumulation curves [25] of cumulative parasite species richness against sampling events for each primate species (*N* = 41) and country (*N* = 21) with 30 or more sampling events. To reduce the effects of inter-sampling event heterogeneity on the shapes of the curves, we used rarefaction (Supplementary Data, Appendix 1) to produce smooth mean species accumulation curves, with confidence intervals 2 standard deviations from the mean.

Next, we used the data from our curves to predict parasite species richness for these 41 primates and 21 countries. We used two nonparametric algorithms, Chao2 and first-order Jackknife (Jackknife1), which have been recommended in this context [25–27] (Supplementary Data, Appendix 1). We also estimated standard errors for our extrapolated parasite species richness values based on references in Oksanen *et al.* [24], and used these to calculate upper and lower bounds on extrapolated parasite species richness. Finally, we plotted species accumulation curves of cumulative parasite species richness for all primate species combined, first using all parasites and then using arthropods, helminths, protozoa and viruses separately. We did not use bacteria and fungi as we had very few of these parasites in our dataset (bacteria = 32 species; fungi = 4 species). 

We used R version 2.15.0 [28] for all the analyses above.

## RESULTS

### Variation in sampling effort among primate species

**Figure 1.**
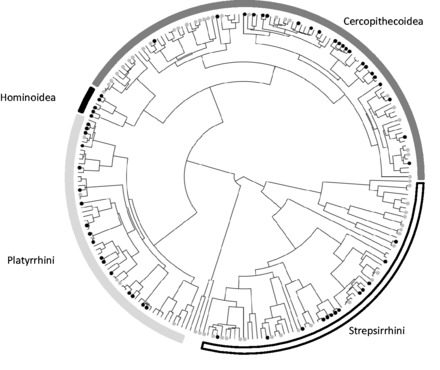
Sampling effort for parasites across the primate phylogeny, assuming that primates should be sampled in proportion to their geographic range size. Species names have been omitted for clarity (see Supplementary Fig. S1 for a larger version with species names). Relative sampling effort was quantified using the residuals from a generalized linear model of ln(geographic range size) against the number of sampling events for each primate species. Gray circles indicate primates with poor sampling relative to their geographic range size (lower 25% of model residuals), black circles indicate primates with better sampling relative to their geographic range size (upper 25% of model residuals).

Sampling effort was unevenly distributed among primates and ranged from 0 (62 ‘unsampled’ species) to 630 sampling events (*P. troglodytes*), with a mean of 30.71 ± 4.970. We plotted sampling effort in relation to the primates' geographic range sizes (Fig. 1 and Supplementary Fig. S1). As predicted, we found that the Hominoidea (great apes) were relatively well sampled in relation to their geographic range size and were sampled significantly more than other primates (*F*_1,225_ = 12.01, *P* = 0.002). We also found great heterogeneity in the degree of parasite sampling across all other major groups of primates, i.e. Old World monkeys (Cercopithecoidea), New World monkeys (Platyrrhini) and strepsirrhines (Strepsirrhini, i.e. lemurs and galagos).

### Variation in sampling effort among geographic regions

**Figure 2.**
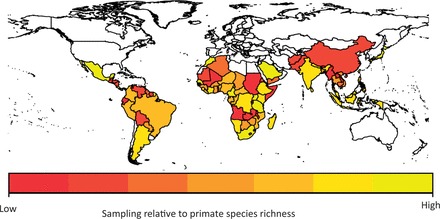
Sampling effort for parasites across the world, assuming that countries should be sampled in proportion to their primate species richness. Relative sampling effort was quantified using the residuals from a generalized linear model of ln(primate species richness) against the number of sampling events for each country. The colors indicate whether countries are poorly sampled (low; red) or better sampled (high; yellow) relative to their primate species richness.

Sampling effort was also unevenly distributed geographically and ranged from 0 (32 ‘unsampled’ countries) to 416 sampling events (Uganda), with a mean of 42.70 ± 9.131. Many countries were poorly sampled in relation to their primate species richness (Fig. 2), with particularly low levels of sampling in parts of South East Asia (including China, Thailand, Cambodia, Myanmar, Laos and Vietnam), Central and Western Africa (including Sudan, Somalia, Angola, Zambia, Guinea and Ghana) and South America (including Bolivia, Ecuador, Venezuela, Guyana and Suriname).

### Modeling variation in sampling effort among primate species

Sampling effort for primate parasites covaried with primate geographic range size, body mass and substrate use: the most sampled primates tend to have larger geographic ranges, to be larger in body mass and to be more terrestrial (Table 1 and Supplementary Table S1). We found no significant effect of phylogenetic distance between humans and primates, indicating that both our close relatives and more distantly related species show evidence for sampling gaps. Our overall model explained around a third of the variation in sampling effort (*r*^2 ^= 0.333), most of which appears to relate to the geographic range size and terrestriality of the primates (in single predictor models, geographic range size: *r*^2 ^= 0.175; body size: *r*^2 ^= 0.061; substrate use: *r*^2 ^= 0.163).

**Table 1.** PGLS model for explaining variation in sampling effort among primate species

Variable	Slope ± SE	*t*_201_
Geographic range size (km^2^)	0.347 ± 0.056	6.222***
Phylogenetic distance (My)	0.189 ± 0.729	0.260
Substrate use	−0.864 ± 0.155	−5.572***
Body size (g)	0.409 ± 0.158	2.597*

*λ* = 0.322; *r*^2 ^= 0.333. Phylogenetic distance is measured as phylogenetic distance from humans in millions of years. Substrate use is a four-state-ordered variable ranging from fully terrestrial to fully arboreal, with more arboreal species scored higher. *P < 0.05; ***P < 0.001.

The number of sampling events for primates showed significant, but intermediate, levels of phylogenetic signal (*N* = 228, *λ* = 0.589). This value was significantly different from both *λ* = 0 and *λ* = 1 (*P* < 0.001), indicating moderate phylogenetic non-independence.

### Modeling geographic variation in sampling effort

Predictably, sampling effort across countries increased with primate species richness. However, neither GDP nor airport density significantly predicted sampling effort (Table 2 and Supplementary Table S2).

**Table 2.** Spatial GLS model with an exponential correlation structure, explaining variation in sampling effort among countries

Variable	Slope ± SE	*t*_84_
Primate species richness	1.241 ± 0.290	4.273***
GDP per capita (USD)	0.437 ± 0.246	1.778
Airport density (airport/km^2^)	−3.559 ± 3.041	−1.170

*ρ* = 1.938; GDP = gross domestic product; ***P < 0.001.

### Extrapolating parasite species richness for primates and countries

**Figure 3.**
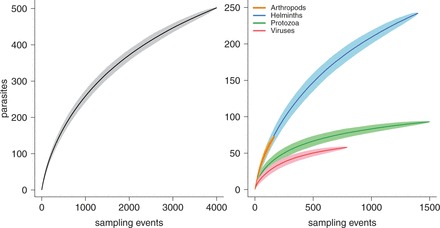
Parasite species accumulation curve for all 161 primates combined and all parasites (left-hand side). Parasite species accumulation curve for all 161 primates combined and helminths, protozoa and viruses separately (right-hand side). Parasites = cumulative parasite species richness. Arthropods = orange curve; helminths = blue curve, protozoa = green curve and viruses = red curve. For each curve, the darker line shows the mean curve and the lighter shaded region shows 2 standard deviations from the mean curve, each obtained from 1000 random permutations of the data. Note that the axes sizes are different on the left- and right-hand plots.

Figure 3 shows the parasite species accumulation curve for all primates combined, and for arthropods, helminths, protozoa and viruses. Across primates and countries, most parasite species accumulation curves were starting to show some downward curvature, indicating a declining rate of parasite species discovery, but in no cases had they approached an asymptote. Interestingly, the different types of parasites accumulated at different rates, with arthropods and helminths accumulating at a much faster rate than protozoa and viruses (see slope differences in Fig. 3). The parasite species accumulation curve for *P. troglodytes* is shown in Supplementary Fig. S2.

Our estimates of parasite species richness using Chao2 and Jackknife1 are shown in Supplementary Tables S3–S5. Using Jackknife1, which appears to give more reasonable values, across the 41 best sampled primates, on average, we predict that there should be between 38 and 79% more parasites than currently recorded in the GMPD (Supplementary Table S3). For countries, on average, we predict that there should be between 29 and 40% more parasites than currently recorded in the GMPD (Supplementary Table S4). For all 161 primates in our study combined, we should find between 685 and 713 parasites, i.e. between 36 and 42% more parasites than the 502 parasites identified to species level that are currently reported in the GMPD for these 161 primates (Supplementary Table S5).

## DISCUSSION

### Variation in sampling effort among primate species and geographic regions

Virtually every broadscale comparison of sampling effort, whether sampling disease agents in epidemiology or species in biodiversity studies, reveals bias in what is sampled. In epidemiology, for example, sampling may be highest for diseases with easily detectable symptoms and for areas easily accessed by medical personnel. Here, we showed that primate parasites are also unevenly sampled across both primate species and space. This supports previous studies of sampling gaps in primate parasites that used an earlier version of the GMPD data [13], but unlike previous studies, we also investigated the drivers of sampling effort variation across primates and geographic areas.

For our analyses of variation in sampling effort among primate species, we predicted that our closest relatives (chimpanzees, gorillas and orangutans) would be relatively well sampled because a great deal of research has focused on these species. We expected most other primate species to be comparatively poorly sampled, except when they are more terrestrial or larger in body mass. As predicted, chimpanzees, gorillas and orangutans were generally better sampled. However, we found incredible variation in sampling among all other major primate groups, intermediate phylogenetic signal in sampling effort and no significant relationship between sampling effort and the phylogenetic distance from humans to the primate in question. Instead, our models suggested that most variation in sampling effort among primates can be explained by the geographic range size and level of terrestriality of the primates. Put more simply, the primates that researchers sample most are the species they encounter most often, including those that are more likely to be on the ground than in the trees. This is also supported by the low sampling of nocturnal primates.

However, our models only explained 30% of the variation in sampling effort across primates, indicating that we did not capture every explanation for this variation. Some primates may be sampled because they are already intensively studied for infectious disease, with researchers building on previous knowledge rather than starting from scratch. Other species may be sampled thoroughly because they live in frequently used and well-equipped field sites. Some of the variation in sampling may have more idiosyncratic explanations; for example, the extensive sampling of some *Macaca* species likely reflects their use in medical research.

We also identified great heterogeneity in sampling among countries, even among those in the same region. We found particularly low sampling in parts of South East Asia, Central and Western Africa, and South America, and better sampling in Eastern Africa and Brazil. However, the only variable in our statistical models that predicted sampling effort among countries was the primate species richness of the country, with parasite sampling highest in countries that have more primates to sample. We expected that the GDP of the countries would also positively affect sampling effort, but we found no evidence for this in our analyses, possibly because much of the research is not funded by the country in which the research takes place. In fact, on average, only 22% of tropical biological field station funding comes from the host country [29]. Perhaps a better predictor of sampling effort would be the number of research stations in a country.

Our parasite species accumulation curves, for both primate species and countries, were starting to show some downward curvature, but in no cases had they approached an asymptote. In these analyses, we only used species or countries with at least 30 sampling events. This indicates that, at least for these fairly well-sampled primates and countries, sampling is slowly approaching levels sufficient to quantify parasite species richness. However, when we extended these analyses to extrapolate parasite species richness values, we found that even within our best sampled primates and countries, we are missing substantial parasite diversity. On average, we predicted that 38–79% more parasite species than currently reported in the GMPD should be found in our best sampled primate species and 29–40% more parasite species than currently reported in the GMPD should be found in our best sampled countries. This emphasizes exactly how poor our sampling is across all primates and countries. The other primates and countries obviously represent even larger gaps in our knowledge.

Sampling was also uneven across types of parasites; when we analyzed arthropods, helminths, protozoa and viruses separately, we saw faster rates of parasite accumulation in arthropods and helminths but with little evidence for sufficient sampling in these species. This is interesting given that helminths make up 48% of the parasite species in our study (arthropods = 33%; bacteria = 6%; fungi = 0.8%; protozoa = 19% and viruses = 12%). We were not able to fit species accumulation curves to bacteria or fungi because we have so few bacteria and fungi species in our dataset. Given the importance of bacterial and fungal emerging diseases in humans [2, 30], this lack of sampling in wild primates is of concern. Another concern is that although viruses make up only 12% of the parasites in our dataset, viruses arguably present the greatest zoonotic disease threat to humans because their fast rates of evolution will allow them to easily adapt to new hosts [3, 5]. The relatively low number of viruses probably reflects detection bias. Viruses are very hard to detect, and even when detected prove difficult to classify. Therefore, our results probably grossly underestimate the number of viruses present in primates.

### Priorities for future research

Identifying parasite sampling gaps across primate species and geographic regions is only the first step, we need to find strategies to minimize these sampling gaps if we are to predict which primate infectious diseases may emerge in humans. One solution is to set research priorities based on the sampling gaps [13], for example, by focusing effort and funding on relatively poorly sampled primate species, arboreal primates, those with small geographic ranges or those found in relatively poorly sampled regions of South East Asia, Central and Western Africa, and South America.

Focusing on relatively poorly sampled primate species and areas may improve our general understanding of primate parasites, but it is only one factor in predicting risk to humans. For example, hosts are more likely to share parasites with their close relatives than with more distant relatives [9, 10]. Thus, continuing to focus our sampling efforts on parasites of our closest relatives (chimpanzees, gorillas and orangutans) may provide the greatest return in the case of risks to humans. This is particularly important because we found that chimpanzees are expected to have 33–50% more parasites than currently found in the GMPD. In addition, ecological similarities also influence parasite sharing among primates, and humans share more parasites with terrestrial than arboreal primate species [9, 10]. As with sampling effort, this probably reflects higher contact rates among humans and terrestrial primates compared with arboreal primates. As a related issue, a host living at higher density is expected to have higher prevalence of parasites and may have more contact with human populations or our domesticated animals, thus increasing opportunities for host shifts to humans. The large numbers of zoonotic emerging infectious diseases with rodent or domesticated animal sources also highlight the importance of rates of contact and host density for disease emergence in humans [2, 3, 5].

## CONCLUSIONS AND IMPLICATIONS

The aim of this study was to identify where the gaps lie in our knowledge of primate parasites. We found that sampling effort was unevenly distributed across primate species and countries, and that the best predictors of sampling effort were the geographic range size or terrestriality of the primate species, or the primate species richness of the country. We also found that, according to our extrapolations of parasite species richness, even our best sampled primates and countries were still vastly under-sampled, typically with only a quarter to two-thirds of their parasites documented, and possibly even less given that fungi and bacteria are so under-represented in current records. This implies that if we want to predict primate disease emergence in humans, more sampling for parasites is needed across all primate species and countries. This is especially important as human populations grow and spread into new areas where they will encounter more primates and consequently more diseases.

## SUPPLEMENTARY DATA

Supplementary data is available at *EMPH* online and the Dryad repository: doi:10.5061/dryad.510sb.

**Conflict of interest**: none declared.

Supplementary Data
