# Supplementary material for: Identifying future zoonotic disease threats: Where are the gaps in our understanding of primate infectious diseases?
Source: Evol Med Public Health. 2013 Jan 22;2013(1):27–36. doi: 10.1093/emph/eot001 (PMC3868449; doi:10.1093/emph/eot001)
Supplement: Supplementary Data [file supp_2013_1_27__index.html]

Identifying future zoonotic disease threats — Supplementary Data 

# Identifying future zoonotic disease threats

## Supplementary Data

files

**Files in this Data Supplement:**

- Supplementary Data - zip file
