# Supplementary material for: Identifying future zoonotic disease threats: Where are the gaps in our understanding of primate infectious diseases?
Source: Evol Med Public Health. 2013 Jan 22;2013(1):27–36. doi: 10.1093/emph/eot001 (PMC3868449; doi:10.1093/emph/eot001)
Supplement: Supplementary Data [file supp_eot001_suppl_data.zip › Appendix_3-Supplementary_figure_legends_Dec12.docx]

**Appendix 3: Supplementary figure legends**

**Figure S1**: Sampling effort for parasites across the primate phylogeny, assuming that primates should be sampled in proportion to their geographic range size. Well- and poorly-sampled primates were identified using the residuals from a generalised linear model of ln(geographic range size) against the number of sampling events for each primate species. Blue tip labels indicate primates with fewer sampling events than expected given their geographic range size (poorly-sampled; lower 25% of model residuals), red tip labels indicate primates with more sampling events than expected given their geographic range size (well-sampled; upper 25% of model residuals).

**Figure S2:** Parasite species accumulation curve for *Pan troglodytes* (chimpanzee). Parasites = cumulative parasite species richness. The black line shows the mean curve, and the gray shaded region shows two standard deviations from the mean curve, each obtained from 1000 random permutations of the data.
