# Supplementary material for: Identifying future zoonotic disease threats: Where are the gaps in our understanding of primate infectious diseases?
Source: Evol Med Public Health. 2013 Jan 22;2013(1):27–36. doi: 10.1093/emph/eot001 (PMC3868449; doi:10.1093/emph/eot001)
Supplement: Supplementary Data [file supp_eot001_suppl_data.zip › Appendix_1-Supplementary_methods_Dec12.docx]

**Appendix 1: Supplementary methods**

**ANALYSES**

*Variation in sampling effort among primate species*

We estimated sampling relative to geographic range size using the residuals from a phylogenetic generalized least squares (PGLS) model of ln(sampling events) against ln(geographic range size). We used PGLS models to deal with the potential statistical non-independence introduced because primate species have inherited similarities from their common ancestors, rather than through independent evolution [[1](#_ENREF_1), [2](#_ENREF_2)]. PGLS is based on the usual generalized least squares (GLS) model except that the phylogenetic dependence of the data is incorporated into the structure of the error term [[3-5](#_ENREF_3)]. This error term consists of a matrix of expected trait covariances calculated using the phylogeny and the maximum likelihood (ML) estimate of λ. The parameter λ is a multiplier of the off-diagonal elements of a phylogenetic variance-covariance matrix that best fits the data, and varies between λ = 1, where the data are structured according to a Brownian motion model of trait evolution, and λ = 0, where the data have no phylogenetic structure [[4](#_ENREF_4), [6](#_ENREF_6)]. For each regression, λ is estimated for the residual error term [[7](#_ENREF_7)], along with the other regression parameters. Thus regressions are carried out whilst controlling for the actual degree of phylogenetic non-independence present.

We expected great apes (Hominoidea) to be better sampled than other primates, so we tested this using phylogenetic analysis of variance (ANOVA) using the R package geiger [[8](#_ENREF_8)]. Phylogenetic ANOVAs are the same as non-phylogenetic ANOVAs, except that the p value is estimated using simulations following the method of [[9](#_ENREF_9)]. First the F statistic for the analysis is estimated, and then the null distribution of this F statistic is obtained by simulating new sets of dependent variables on the phylogenetic tree. Simulations are run under a Brownian motion model with the Brownian rate parameter (σ^2^) estimated from the average squared independent contrast.

*Variation in sampling effort among geographic regions*

We used the R packages sp [[10](#_ENREF_10)], rgeos [[11](#_ENREF_11)] and rworldmap [[12](#_ENREF_12)] to extract the number of sampling events, sampled and “unsampled” primate species, and overall primate species richness within each country. Next, following a similar method to that used above, we estimated sampling relative to primate species richness within each country using the residuals from a spatial generalized least squares (GLS) model of ln(sampling events) against ln(primate species richness). We used spatial GLS models to deal with potential spatial autocorrelation in our models introduced because countries in close proximity are likely to have similar values for primate species richness. Spatial GLS models are similar to those used for phylogenetic autocorrelation above, but instead of incorporating phylogenetic information we incorporated the spatial structure of the data into the error term. This error term consists of a matrix of expected spatial covariances calculated using the latitudinal and longitudinal centroids of each country, the maximum likelihood estimate of *ρ* (in degrees) and assuming an exponential correlation structure (i.e., variable values get exponentially less similar with increasing distance) [[13](#_ENREF_13)]. The parameter *ρ* is the maximum geographic distance over which spatial autocorrelation occurs, and is the equivalent of λ in a PGLS.

*Extrapolating parasite species richness for primates and countries*

We used functions from the R package vegan [[14](#_ENREF_14)] to extrapolate parasite species richness for primates and countries. First we used species accumulation curves which plot the accumulation of new species relative to some measure of sampling effort [[15](#_ENREF_15), [16](#_ENREF_16)]. If these curves asymptote at the observed number of species it indicates that the sampling effort is sufficient to detect all parasite species present [[15](#_ENREF_15)]. Here, our measure of sampling effort is the number of sampling events and our species accumulation curves therefore show how cumulative parasite species richness increases with the number of sampling events. We plotted species accumulation curves of cumulative parasite species richness for each primate species (N = 41) and country (N = 21) with 30 or more sampling events. To reduce the effects of inter-sampling event heterogeneity on the shapes of the curves, we used rarefaction as follows. For a given primate or country, we randomly selected one sampling event and defined the cumulative parasite species richness as one. We then randomly selected a second sampling event and if the parasite for that sampling event was not the same as that found in the first sampling event, we updated the cumulative parasite species richness to two. If the parasite for that sampling event was not the same as that found in the first sampling event, the cumulative parasite species richness remained at one. This random sampling (without replacement) continued until all sampling events were counted. The process was then repeated 1000 times so that a smooth mean species accumulation curve, with confidence intervals two standard deviations from the mean, was produced.

Next, we used the data from our species accumulation curves to predict parasite species richness for each primate species (N = 41) and country (N = 21) with 30 or more sampling events. We used two nonparametric algorithms, Chao2 and first order Jackknife (Jackknife1), which have been recommended for extrapolating parasite species richness [[15](#_ENREF_15), [17](#_ENREF_17), [18](#_ENREF_18)]. Both of these algorithms produce parasite species richness estimates based on the number of rarely sampled parasite species, using the assumption that if there are some rarely sampled species in the primate or country, there are probably more parasite species that have not yet been sampled. Chao2 and Jackknife1 are calculated as follows:

Chao 2: *Extrapolated parasite species richness =* $S_{0}+ {{a_{1}}^{2}}/\left( 2*a_{2} \right)$ (3)

Jackknife1: *Extrapolated parasite species richness =* $S_{0}+ a_{1}* \left( {N-1}/N \right)$ (4)

where *S_0_* is the observed number of parasite species in the primate species or country, *a_1_* and *a_2_* are the number of parasite species occurring only in one (*a_1_*) or only in two (*a_2_*) sampling events in the primate species or country, and *N* is the number of sampling events in the primate species or country. We also estimated standard errors for our extrapolated parasite species richness values based on [[14](#_ENREF_14), [19](#_ENREF_19), [20](#_ENREF_20)], and used these to calculate upper and lower bounds on extrapolated parasite species richness. Finally, we plotted species accumulation curves of cumulative parasite species richness for all primate species combined, first using all parasites and then using arthropods, helminths, protozoa and viruses separately. We did not plot parasite species accumulation curves for bacteria and fungi separately as we had very few of these parasites in our dataset (bacteria = 32 species; fungi = 4 species).

**References**

1. Harvey PH, Clutton-Brock TH. Life history variation in primates. *Evolution* 1985; **39**: 559–81.

2. Harvey PH, Pagel MD. *The comparative method in evolutionary biology*. Oxford: Oxford University Press, 1991.

3. Freckleton RP, Harvey PH, Pagel M. Phylogenetic analysis and comparative data: a test and review of evidence. *Am Nat* 2002; **160**: 712-26.

4. Pagel M. Inferring the historical patterns of biological evolution. *Nature* 1999; **401**: 877-84.

5. Rohlf FJ. Comparative methods for the analysis of continuous variables: geometric interpretations. *Evolution* 2001; **55**: 2143-60.

6. Pagel M. Inferring evolutionary processes from phylogenies. *Zool Script* 1997; **26**: 331–48.

7. Revell LJ. Phylogenetic signal and linear regression on species data. *Methods Ecol Evol* 2010; **1**: 319-29.

8. Harmon LJ, Weir JT, Brock CD *et al.* GEIGER: investigating evolutionary radiations. *Bioinformatics* 2008; **24**: 129-31.

9. Garland T, Dickerman AW, Janis CM *et al.* Phylogenetic analysis of covariance by computer-simulation. *Syst Biol* 1993; **42**: 265-92.

10. Pebesma EJ, Bivand RS. sp: classes and methods for spatial data in R. *R News* 2005; **5**.

11. Bivand R, Rundel C. rgeos: Interface to Geometry Engine - Open Source (GEOS). R package version 0.2-7. <http://CRAN.R-project.org/package=rgeos>. 2012.

12. South A, Scutt-Phillips J, Rowlingson B *et al.* rworldmap: for mapping global data. R package version 0.1310. <http://CRAN.R-project.org/package=rworldmap>. 2012.

13. Dormann CF. Effects of incorporating spatial autocorrelation into the analysis of species distribution data. *Glob Ecol Biogeog* 2007; **16**: 129-38.

14. Oksanen J, Blanchet FG, Kindt R *et al.* vegan: community ecology package. R package version 2.0-3. 2012.

15. Dove ADM, Cribb TH. Species accumulation curves and their applications in parasite ecology. *Trends Parasit* 2006; **22**: 568-74.

16. Colwell RK, Coddington JA. Estimating terrestrial biodiversity through extrapolation. *Phil Trans Roy Soc B* 1994; **345**: 101-18.

17. Poulin R, Krasnov BR, Mouillot D. Host specificity in phylogenetic and geographic space. *Trends Parasit* 2011; **27**: 355-61.

18. Walther BA, Morand S. Comparative performance of species richness estimation methods. *Parasitology* 1998; **116**: 395-405.

19. Smith EP, van Belle G. Nonparametric estimation of species richness. *Biometrics* 1984; **40**: 119-29.

20. Chao A. Estimating the population size for capture-recapture data with unequal catchability. *Biometrics* 1987; **43**: 783-91.
