# Supplementary material for: Identifying future zoonotic disease threats: Where are the gaps in our understanding of primate infectious diseases?
Source: Evol Med Public Health. 2013 Jan 22;2013(1):27–36. doi: 10.1093/emph/eot001 (PMC3868449; doi:10.1093/emph/eot001)
Supplement: Supplementary Data [file supp_eot001_suppl_data.zip › Appendix_2-Supplementary_tables_Dec12.docx]

**Appendix 2: Supplementary tables**

Table S1: Linear model (OLS) for explaining variation in sampling effort among primate species. r^2^ = 0.358. Phylogenetic distance is measured as phylogenetic distance from humans in millions of years. Substrate use is a four-state ordered variable ranging from fully terrestrial to fully arboreal, with more arboreal species scored higher. *p < 0.05; ***p < 0.01; ***p < 0.001.

| **Variable** | **slope ± SE** | **t_201_** |
| --- | --- | --- |
| Geographic range size (km^2^) | 0.365 ± 0.052 | 7.065*** |
| Phylogenetic distance (My) | 0.881 ± 0.430 | 2.050* |
| Substrate use | -0.719 ± 0.139 | -5.186*** |
| Body size (g) | 0.361 ± 0.129 | 2.801** |

Table S2: Linear model (OLS) explaining variation in sampling effort among countries. r^2^ = 0.219. GDP = gross domestic product; ***p < 0.001.

| **Variable** | **slope ± SE** | **t_84_** |
| --- | --- | --- |
| Primate species richness | 1.259 ± 0.281 | 4.488*** |
| GDP per capita (USD) | 0.406 ± 0.234 | 1.734 |
| Airport density (airport/km^2^) | -2.743 ± 3.084 | -0.889 |

Table S3: Lower and upper estimates of parasite species richness for primates with more than 30 sampling events, estimated using Chao2 or Jackknife1. PSR = parasite species richness.

|  |  |  | **PSR Chao2** | | **PSR Jackknife1** | |
| --- | --- | --- | --- | --- | --- | --- |
| **Primate species** | **Sampling occasions** | **PSR observed** | **lower** | **upper** | **lower** | **upper** |
| *Alouatta caraya* | 56 | 21 | 24.73 | 101.77 | 30.23 | 37.31 |
| *Alouatta guariba* | 31 | 13 | 3.42 | 122.58 | 19.62 | 25.74 |
| *Alouatta palliata* | 90 | 21 | 25.69 | 58.56 | 30.29 | 37.42 |
| *Alouatta pigra* | 50 | 4 | 4.00 | 4.00 | 4.57 | 7.35 |
| *Alouatta seniculus* | 52 | 33 | 60.33 | 161.92 | 52.62 | 62.42 |
| *Aotus trivirgatus* | 38 | 12 | 11.51 | 44.49 | 17.04 | 22.54 |
| *Arctocebus calabarensis* | 30 | 2 | 2.00 | 2.00 | 2.57 | 5.30 |
| *Callithrix jacchus* | 38 | 17 | 17.87 | 29.63 | 22.84 | 28.68 |
| *Cebus apella* | 70 | 15 | 14.89 | 31.44 | 19.29 | 24.51 |
| *Cebus capucinus* | 43 | 20 | 22.37 | 41.83 | 27.50 | 33.98 |
| *Cercocebus torquatus* | 37 | 5 | 1.34 | 24.66 | 6.95 | 10.84 |
| *Cercopithecus ascanius* | 73 | 20 | 20.76 | 69.24 | 26.74 | 32.98 |
| *Cercopithecus cephus* | 36 | 6 | 1.36 | 35.64 | 8.69 | 13.04 |
| *Cercopithecus mitis* | 35 | 18 | 18.74 | 28.83 | 23.83 | 29.66 |
| *Cercopithecus mona* | 44 | 9 | 3.38 | 50.62 | 12.47 | 17.26 |
| *Cercopithecus nictitans* | 37 | 7 | 5.70 | 20.80 | 9.69 | 14.04 |
| *Chlorocebus aethiops* | 100 | 23 | 28.24 | 56.96 | 33.16 | 40.56 |
| *Colobus guereza* | 49 | 16 | 5.42 | 147.58 | 23.53 | 30.02 |
| *Erythrocebus patas* | 36 | 10 | 9.28 | 19.05 | 12.69 | 17.04 |
| *Gorilla beringei* | 104 | 41 | 51.44 | 78.96 | 58.14 | 67.43 |
| *Gorilla gorilla* | 114 | 44 | 62.92 | 106.08 | 65.61 | 75.91 |
| *Lophocebus albigena* | 30 | 14 | 13.59 | 15.31 | 15.23 | 18.57 |
| *Macaca fascicularis* | 81 | 29 | 32.26 | 49.89 | 38.28 | 45.40 |
| *Macaca fuscata* | 139 | 18 | 18.40 | 38.94 | 23.13 | 28.75 |
| *Macaca mulatta* | 87 | 24 | 25.43 | 39.24 | 30.76 | 37.01 |
| *Macaca sinica* | 39 | 14 | 7.39 | 69.61 | 18.24 | 23.40 |
| *Mandrillus sphinx* | 58 | 16 | 5.42 | 147.58 | 23.55 | 30.07 |
| *Miopithecus talapoin* | 39 | 4 | 4.00 | 4.00 | 4.57 | 7.33 |
| *Pan troglodytes* | 411 | 59 | 76.29 | 119.83 | 78.95 | 88.93 |
| *Papio anubis* | 196 | 50 | 67.29 | 110.83 | 69.90 | 79.85 |
| *Papio cynocephalus* | 110 | 46 | 63.32 | 101.58 | 67.61 | 77.90 |
| *Papio hamadryas* | 47 | 15 | 15.00 | 15.00 | 21.69 | 27.88 |
| *Papio papio* | 32 | 21 | 26.67 | 52.83 | 31.78 | 39.28 |
| *Papio ursinus* | 145 | 57 | 106.57 | 238.63 | 84.97 | 96.56 |
| *Perodicticus potto* | 39 | 6 | 6.00 | 6.00 | 7.95 | 11.85 |
| *Pongo pygmaeus* | 74 | 22 | 22.71 | 30.38 | 28.75 | 34.98 |
| *Saguinus fuscicollis* | 31 | 15 | 16.82 | 38.18 | 21.62 | 27.74 |
| *Saguinus geoffroyi* | 36 | 16 | 19.73 | 96.77 | 25.13 | 32.14 |
| *Saguinus midas* | 57 | 14 | 14.06 | 54.44 | 19.89 | 25.79 |
| *Saimiri sciureus* | 140 | 35 | 43.68 | 90.32 | 46.91 | 54.86 |
| *Trachypithecus cristatus* | 38 | 16 | 17.11 | 35.14 | 21.84 | 27.68 |

Table S4: Lower and upper estimates of parasite species richness for countries with more than 30 sampling events, estimated using Chao2 or Jackknife1. PSR = parasite species richness.

|  |  |  | **PSR Chao2** | | **PSR Jackknife1** | |
| --- | --- | --- | --- | --- | --- | --- |
| **Country** | **Sampling occasions** | **PSR observed** | **lower** | **upper** | **lower** | **upper** |
| Brazil | 229 | 28 | 31.61 | 60.39 | 36.50 | 43.40 |
| Cameroon | 372 | 24 | 26.65 | 51.60 | 31.66 | 38.28 |
| Central African Republic | 30 | 7 | 6.36 | 8.97 | 7.57 | 10.30 |
| Colombia | 77 | 25 | 25.14 | 31.26 | 30.10 | 35.69 |
| Cote d'Ivoire | 34 | 8 | 4.34 | 27.66 | 9.94 | 13.82 |
| Democratic Republic of the Congo | 93 | 47 | 66.00 | 103.00 | 71.26 | 82.10 |
| Ethiopia | 48 | 10 | 9.13 | 12.87 | 10.57 | 13.34 |
| French Guiana | 49 | 22 | 22.53 | 30.47 | 27.88 | 33.76 |
| India | 74 | 19 | 18.54 | 21.46 | 20.97 | 24.92 |
| Indonesia | 44 | 18 | 19.11 | 37.14 | 23.86 | 29.73 |
| Japan | 85 | 14 | 5.41 | 103.59 | 19.93 | 25.86 |
| Kenya | 105 | 34 | 45.95 | 103.05 | 47.63 | 56.03 |
| Madagascar | 82 | 35 | 41.78 | 64.22 | 48.59 | 56.97 |
| Malaysia | 111 | 33 | 41.66 | 69.47 | 47.51 | 56.15 |
| Panama | 207 | 50 | 65.81 | 101.79 | 70.80 | 80.95 |
| Peru | 62 | 17 | 17.06 | 26.74 | 21.28 | 26.49 |
| Senegal | 60 | 11 | 9.85 | 16.65 | 12.25 | 15.65 |
| South Africa | 124 | 48 | 63.65 | 104.35 | 66.95 | 76.67 |
| Sri Lanka | 33 | 11 | 9.70 | 24.80 | 13.68 | 18.02 |
| Uganda | 238 | 44 | 53.38 | 88.62 | 57.70 | 66.15 |
| United Republic of Tanzania | 230 | 34 | 42.00 | 154.00 | 45.95 | 53.91 |

Table S5: Lower and upper estimates of parasite species richness for all primates combined, estimated using Chao2 or Jackknife1. PSR = parasite species richness.

|  |  |  | **PSR Chao2** | | **PSR Jackknife1** | |
| --- | --- | --- | --- | --- | --- | --- |
| **Parasite type** | **Sampling occasions** | **PSR observed** | **lower** | **upper** | **lower** | **upper** |
| All parasites | 3999 | 502 | 674.79 | 755.69 | 684.92 | 712.98 |
| Arthropods | 168 | 73 | 109.48 | 165.83 | 107.39 | 120.12 |
| Helminths | 1399 | 242 | 320.04 | 377.02 | 330.99 | 350.87 |
| Protozoa | 1497 | 93 | 101.02 | 120.25 | 111.19 | 120.78 |
| Viruses | 789 | 58 | 64.42 | 87.71 | 70.86 | 79.10 |
