# Supplementary figures and images for: Identifying future zoonotic disease threats: Where are the gaps in our understanding of primate infectious diseases?
Source: Evol Med Public Health. 2013 Jan 22;2013(1):27–36. doi: 10.1093/emph/eot001 (PMC3868449; doi:10.1093/emph/eot001)

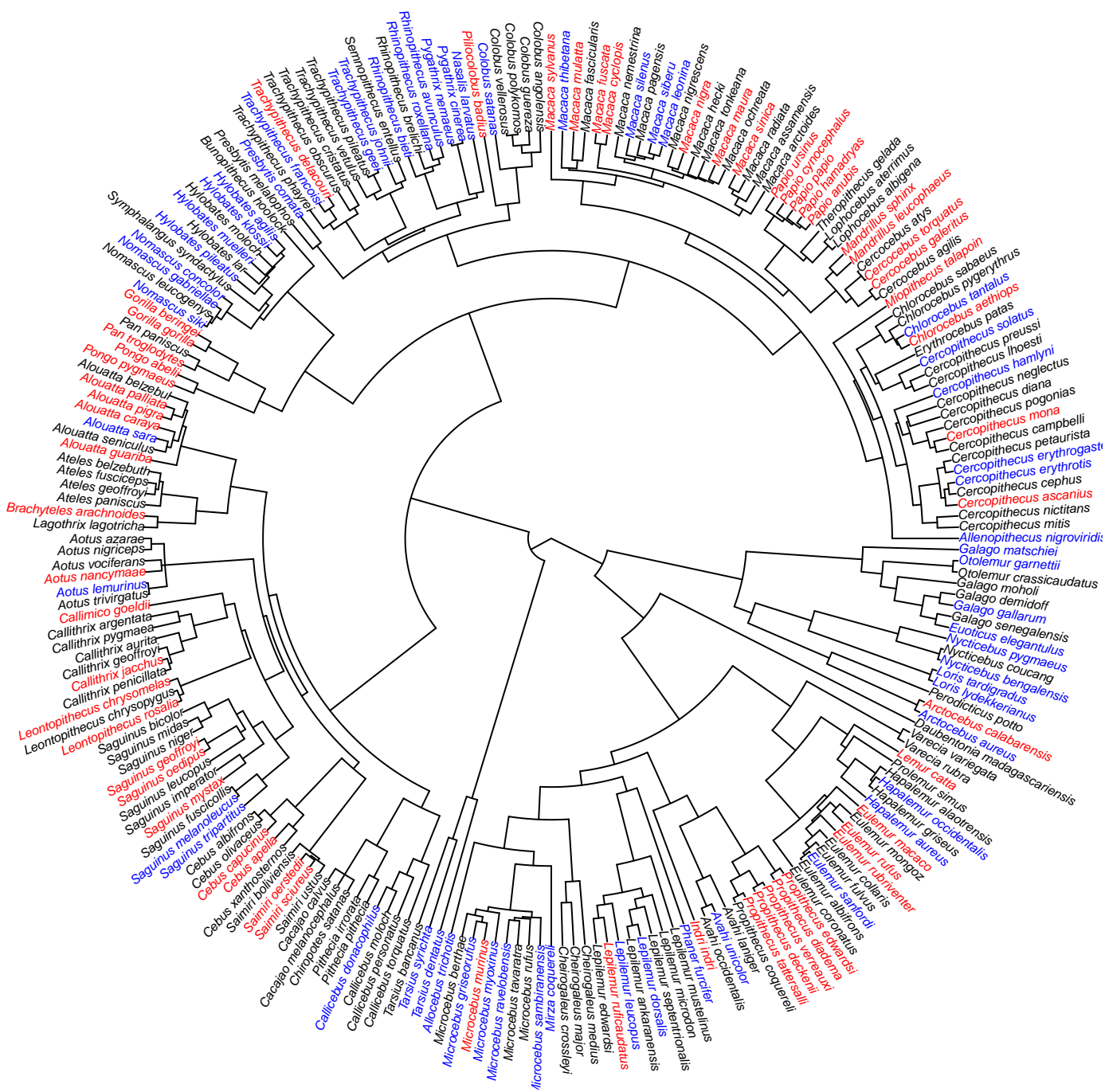

Supplement: Supplementary Data [file supp_eot001_suppl_data.zip › Figure_S1_Oct12.pdf]

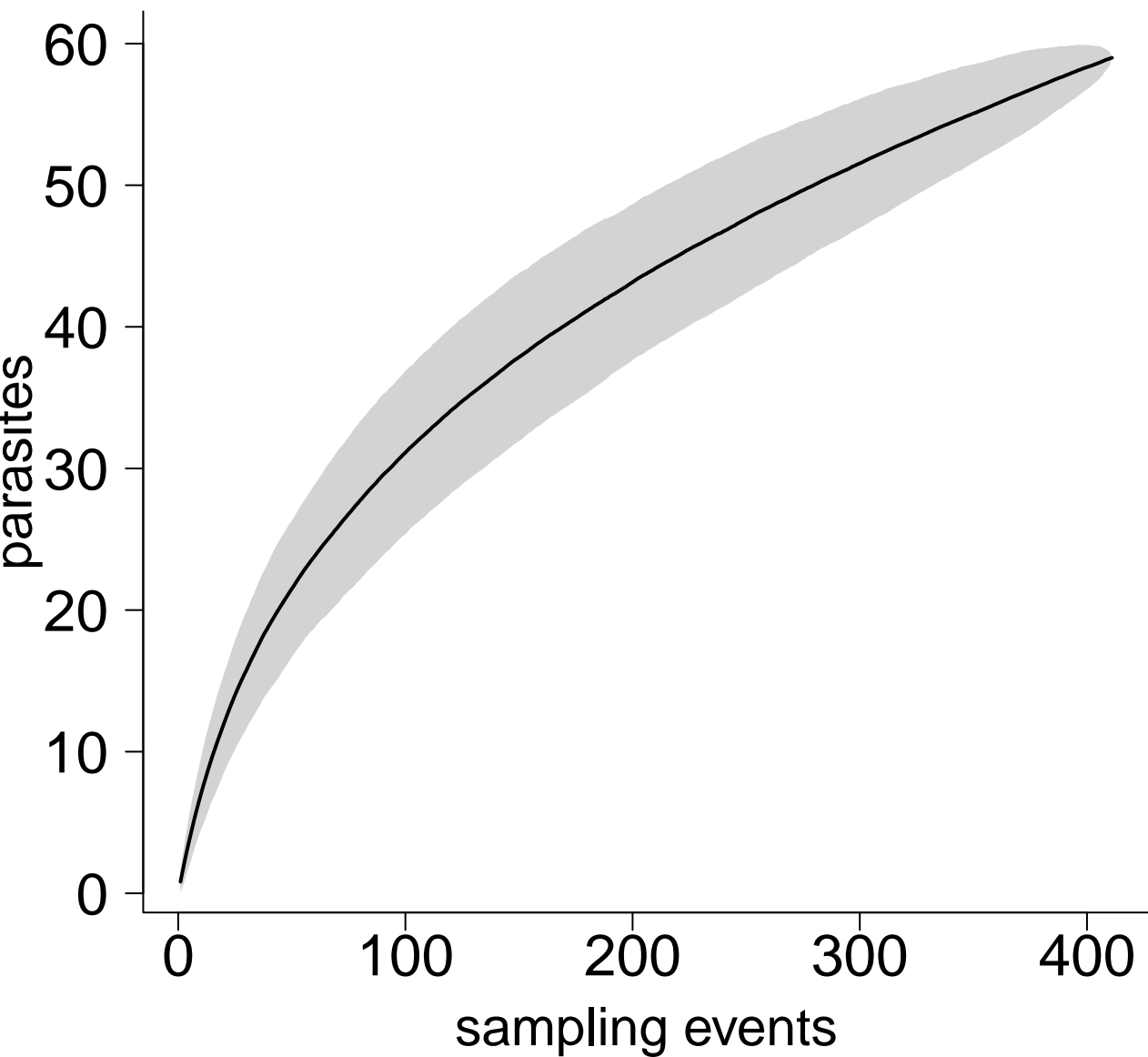

Supplement: Supplementary Data [file supp_eot001_suppl_data.zip › Figure_S2.pdf]
